# Altered Dorsolateral Prefrontal Activation in Response to Verbal Fluency Task and Whole‐Brain Resting‐State Functional Connectivity Strength in Acute Carbon Monoxide Poisoning

**DOI:** 10.1002/brb3.71536

**Published:** 2026-06-12

**Authors:** Jian‐Qing Pan, Qun‐Jun Liang, Ming‐Xing Tang, Lian Duan, Qing‐Ping Zhang

**Affiliations:** ^1^ Department of Hyperbaric Oxygen Affiliated Nanshan Hospital of Shenzhen University Shenzhen Guangdong China; ^2^ Department of Hyperbaric Oxygen Shenzhen Nanshan People's Hospital Shenzhen Guangdong China; ^3^ School of Marxism Guangxi University of Science and Technology Liuzhou Guangxi China; ^4^ Department of Radiology Shenzhen Nanshan People's Hospital Shenzhen Guangdong China; ^5^ Department of Otorhinolaryngology Shenzhen Nanshan People's Hospital Shenzhen Guangdong China; ^6^ Shenzhen Key Laboratory of Affective and Social Neuroscience, Center for Brain Disorders and Cognitive Sciences Shenzhen University Shenzhen Guangdong China; ^7^ Department of Neurosurgery Affiliated Nanshan Hospital of Shenzhen University Shenzhen Guangdong China

**Keywords:** carbon monoxide poisoning, dorsolateral prefrontal cortex, functional connectivity change, functional near‐infrared spectroscopy, resting‐state functional connectivity, verbal fluency task

## Abstract

**Objective:**

The study aims to investigate changes in brain activity during the verbal fluency task (VFT) and resting‐state brain network features in patients with acute carbon monoxide (CO) poisoning using functional near‐infrared spectroscopy (fNIRS). We also explored the relationship between cognitive function and the fNIRS‐derived features of oxyhemoglobin (Oxy‐Hb) concentration curves in the cerebral cortex.

**Methods:**

We first performed a cross‐sectional study in patients with acute CO poisoning and healthy controls between January and December 2024. The fNIRS‐derived features (centroid and mean) of the Oxy‐Hb curve in the frontotemporal cortex during VFT were compared between CO poisoning patients and healthy controls.

The functional connectivity of brain networks in patients with acute CO poisoning during the resting state was also analyzed to identify factors representing network features of the cerebral cortex in patients with acute CO poisoning.

We then performed a prospective cohort study to compare the brain activity changes after the hyperbaric oxygen therapy in patients with acute CO poisoning.

**Results:**

The study included 29 patients with CO poisoning and 28 healthy controls. During the VFT, the centroid of the Oxy‐Hb curve in Brodmann area 46 was significantly lower in CO poisoning patients compared to the healthy controls (*p* < 0.01). The centroid and mean values of Brodmann area 46 exhibited a significant positive correlation with Mini‐Mental State Examination scores, while the mean value was positively correlated with VFT scores. Mean values in Brodmann areas 11 and 38 were significantly lower in CO poisoning patients (both *p* < 0.05). The average whole‐brain resting‐state functional connectivity strength (FCS) was significantly lower in CO poisoning patients compared to healthy controls (*p* < 0.05), with significant differences in FCS observed in 39 pairs of channels (*p* < 0.01). Twenty sessions of hyperbaric oxygen therapy resulted in improved FCS (*p* < 0.01) and centroid values for the Oxy‐Hb curve in the left dorsolateral prefrontal cortex during the VFT (*p* < 0.05).

**Conclusions:**

The centroid values of the Oxy‐Hb curve in the left dorsolateral prefrontal cortex during the VFT, along with the average whole‐brain resting‐state FCS, potentially could serve as characteristic indicators of brain function in patients with acute CO poisoning.

## Graphical abstract

The centroid values of the oxyhemoglobin (Oxy‐Hb) curve in the left dorsolateral prefrontal cortex during the verbal fluency test (VFT) may serve as potential neurofunctional biomarkers in patients with acute carbon monoxide (CO) poisoning.

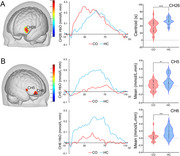

## 1 Introduction

Carbon monoxide (CO) poisoning is the leading cause of death from accidental poisoning in the clinical practice (GBD 2021 Carbon Monoxide Poisoning Collaborators 2023; Cui et al. 2022; Weaver 2009). The central nervous system is particularly susceptible to acute CO poisoning due to its high metabolic and energy demands. In addition to acute neurological symptoms (Guzman 2012), a substantial proportion of survivors develop delayed encephalopathy (DEACMP) after a transient recovery period, leading to persistent cognitive impairments and diminished quality of life (Hu et al. 2011). Despite its clinical significance, the precise neurobiological mechanisms underlying brain dysfunction following acute CO exposure remain incompletely understood.

CO primarily affects the central nervous system. Direct assessment of CO levels in brain tissue would ideally provide the most accurate evaluation of its impact. Normal individuals naturally produce endogenous CO (Maines 1997). An earlier animal study employing a reduction gas analysis failed to detect increased CO levels in rat brain tissue following CO exposure (Cronje et al. 2004). In contrast, a more recent study using the hemoCD assay to measure CO in human tissues found significantly higher CO levels across all brain regions of the exposure group compared with the control group (Mori et al. 2025). However, these CO detection methods depend on histological tissue samples, and no current technology can directly measure CO levels in living brain tissues.

Typically, CO exposure in the body is assessed by measuring carboxyhemoglobin (CO‐Hb) saturation in the blood (Peterson and Stewart 1975). A CO‐Hb level exceeding 3% in nonsmokers or 10% in smokers indicates CO exposure (Hampson and Hauff 2008), with levels of 50% or higher generally considered lethal (Kinoshita et al. 2020). However, CO‐Hb levels often fail to correlate with the presence or severity of initial symptoms or long‐term clinical outcomes. This discrepancy suggests that the pathophysiology of CO poisoning may be driven more by systemic inflammatory responses than by simple tissue hypoxia (Weaver et al. 2007).

Early detection and accurate assessment of brain function are critical for guiding timely interventions and improving long‐term neurological outcomes. However, current understanding of the neurofunctional consequences of CO poisoning is largely derived from structural or metabolic imaging studies. For instance, multimodal‐evoked potentials have been proposed as sensitive indicators of brain dysfunction and predictors of DEACMP (He et al. 1993). More recently, magnetic resonance imaging (MRI)‐based studies have identified cortical thinning (T. Wang et al. 2023), hippocampal mean and apparent diffusion coefficients (Jiang et al. 2020), abnormal functional connectivity across whole‐brain and cognitive‐related key brain regions (Yang et al. 2025), and white matter microstructural disruptions (Chang et al. 2009) in CO‐poisoned patients, some of which have been associated with subsequent cognitive decline. While informative, these findings are primarily structural in nature, offering limited insight into the dynamic functional alterations that drive cognitive and behavioral impairments. Specifically, conventional MRI modalities face several practical constraints: high cost, limited temporal resolution, susceptibility to motion artifacts, and difficulty integrating behavioral paradigms during scanning. These limitations hinder their utility for real‐time, task‐evoked functional assessments—a critical dimension for capturing the integrity of neural circuits engaged in cognitive processes such as executive function, language, and memory.

In this context, functional near‐infrared spectroscopy (fNIRS) offers a compelling alternative, as it can non‐invasively quantify cortical hemodynamic responses by detecting changes in oxyhemoglobin (Oxy‐Hb) and deoxyhemoglobin concentrations, thereby providing a direct surrogate of neural activity with high temporal resolution (Pinti et al. 2020). Importantly, fNIRS is portable, cost‐effective, and compatible with a wide range of cognitive tasks, making it uniquely suited for bedside or outpatient monitoring of dynamic brain function (Notte et al. 2024; Okamoto et al. 2004; Tong and Frederick 2010; Visani et al. 2015).

Among cognitive paradigms, the verbal fluency task (VFT) is widely recognized as a robust probe of prefrontal and temporal lobe function. VFT engages executive processes such as working memory, lexical retrieval, and cognitive flexibility that are frequently affected in CO‐poisoned patients (J. Liu et al. 2025; Zanelli et al. 2010). When combined with fNIRS, the VFT enables quantitative, time‐resolved assessment of cortical activation patterns, capturing both the magnitude (mean Oxy‐Hb) and the temporal dynamics (centroid) of hemodynamic responses. These metrics offer a precise and objective measurement on brain functional vitality, surpassing the descriptive capabilities of static imaging modalities.

In parallel, resting‐state fNIRS provides an evaluation of intrinsic functional connectivity across cortical networks, offering complementary information about the brain's baseline organization and its disruptions following injury (Ferrari and Quaresima 2012; Zhao et al. 2016). Graph‐theoretic analyses of resting‐state data further enable the quantification of network topology, including integration and segregation characteristics that reflect cognitive reserve and vulnerability.

Considering the above strengths, we hypothesized that fNIRS, coupled with both task‐based (VFT) and resting‐state paradigms, could serve as a sensitive tool for detecting functional brain alterations in patients with acute CO poisoning. We further proposed that these alterations would correlate with clinical cognitive performance and respond to therapeutic intervention. To test this hypothesis, we conducted a mixed‐methods study involving patients with acute CO poisoning and matched healthy controls. We specifically aimed to (1) characterize task‐evoked cortical activation patterns during VFT using fNIRS‐derived metrics (centroid and mean Oxy‐Hb); (2) examine resting‐state functional connectivity and network topology; and (3) explore the relationship between these neurophysiological markers and cognitive outcomes, as well as their responsiveness to hyperbaric oxygen therapy (HBOT). By integrating task‐based and resting‐state fNIRS, this study aimed to fill a critical gap in the non‐invasive, functional assessment of CO‐induced brain injury and to identify clinically applicable biomarkers for monitoring disease progression and treatment efficacy.

## 2 Materials and Methods

### 2.1 Study Design

This study utilized a mixed‐methods approach, incorporating a cross‐sectional study with a prospective cohort study, conducted at the Shenzhen Nanshan People's Hospital in China between January 2024 and December 2024. The study protocol was approved by the hospital's Scientific Research Ethics Committee (approval number: ky‐2024‐031702). Written informed consent was obtained from all study participants.

### 2.2 Participant Selection

We prospectively recruited adult patients aged 18–65 years who were admitted within 7 days after acute CO poisoning. Exclusion criteria included (1) hearing impairment or auditory disorders, (2) mental illnesses such as schizophrenia or bipolar disorder, (3) cognitive dysfunction such as Alzheimer's disease, Parkinson's disease, or traumatic brain injury, (4) skull defects, (5) intracranial metal implants or pacemakers, and (6) left‐handedness. Age‐matched healthy adults were also recruited as controls and participated in a cognitive activation task and resting‐state scan.

### 2.3 Study Protocol and Data Collection

Clinical information, including age, sex, length of education, CO exposure time, duration of coma, and CO‐Hb saturation (%), was recorded. Cognitive screening was performed using the Mini‐Mental Status Exam (MMSE) obtained on enrollment. We measured the brain activities at rest and during VFT in CO patients and healthy controls. In addition, we repeated these measurements if patients received HBOT.

#### 2.3.1 Brain Activity Evaluation at Rest

Before the task scan, participants were instructed to remain in a quiet, relaxed state, and to refrain from active cognitive engagement for a minimum of 30 min. The ambient temperature was maintained at a comfortable level. Participants were advised to sit comfortably and focus their gaze on a blank wall directly in front of them. In accordance with modified Beer–Lambert law, cerebral cortex oxy‐Hb and deoxy‐Hb concentrations were measured using infrared light at two wavelengths (760 and 850 nm) with a 48 multichannel fNIRS instrument (NirSmart‐II; Danyang Huichuang Medical Equipment Co., Ltd., Beijing, China). Data were sampled at a frequency of 11 Hz, with a total sampling duration of 6 min.

#### 2.3.2 Brain Activity Evaluation During VFT

**FIGURE 1:**
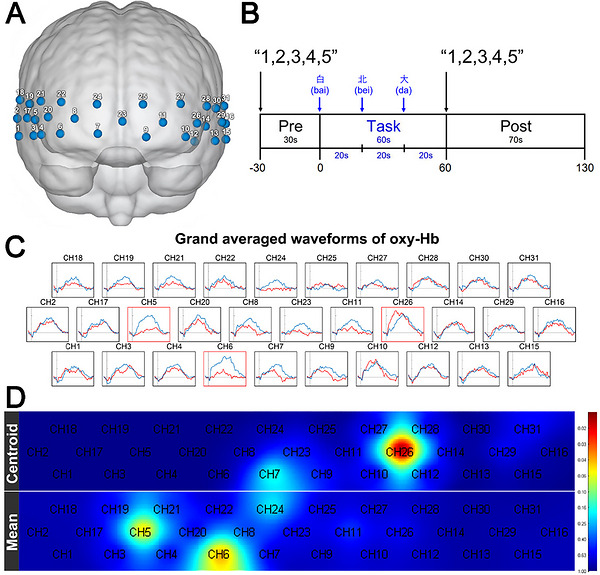
Oxy‐Hb concentration changes during the VFT. (A) Localization of the 31 Oxy‐Hb CHs in the frontotemporal cortex during the VFT. (B) Flow chart of the VFT. (C) Dynamic changes of Oxy‐Hb concentrations across 31 CHs during the VFT. Red represents the patient group; blue represents the control group (data reflect the mean values for all participants in each group). (D) Comparisons between centroid and mean values of the Oxy‐Hb curves from 31 CHs during the VFT between the patient and control groups. CH, channel; Oxy‐Hb, oxyhemoglobin; VFT, verbal fluency task.

Hemoglobin changes in the frontal‐temporal regions were quantified by the NIRS signals recorded during the VFT, which is a neuropsychological test to elicit functional abnormalities relevant to neuropsychiatric disorders (J. Liu et al. 2025; Zanelli et al. 2010). Data were collected using fNIRS instruments (NirSmart‐II, Danyang Huichuang Medical Equipment Co., Ltd., Beijing, China). A frontotemporal cap was used for data collection during the VFT (**Figure** **1A**). The whole test consisted of three stages, with stages 1, 2, and 3 representing the pre‐scan (30 s), task period (60 s), and recovery period (70 s), respectively (**Figure** **1B**). In stages 1 and 3, participants were asked to repeat a list of numbers (“1, 2, 3, 4, and 5”) to rule out the effects of verbal activation on brain activation during stage 2. In stage 2, participants were presented with voice prompts that asked them to generate as many associated words of three Chinese characters ([white], [north], and [big]) as possible within a 15‐s time frame. Participants were instructed to persist in their efforts to form words even if they encountered difficulties in generating new associations during the task. They were also instructed to sit still during the test. For some patients who participated in a second test, three different Chinese characters ([blue], [small], and [golden]) were used.

#### 2.3.3 FNIRS Data Processing

NirSpark software (Danyang Huichuang Medical Equipment Co., Ltd., China) was used to preprocess raw task‐based fNIRS data, which included bad channel (CH) removal, moving average calculation, motion‐related drift correction, and Butterworth filter application to refine cerebral blood oxygen signals(Li et al. 2023). During preprocessing, the raw data were band‐pass filtered between 0.01 and 0.1 Hz to remove physiological noise. Data acquired during the VFT were processed in blocks to compute the activation feature values (including centroid and mean values) for each CH during the VFT. After preprocessing the resting‐state data, a functional connectivity analysis was conducted using NirSpark software to compare differences in connectivity patterns between the patient and control groups.

#### 2.3.4 Resting‐State Brain Network Analysis

To evaluate co‐activation patterns among brain regions during the resting state, we conducted a brain network analysis using data from all fNIRS CHs. The topological properties of the brain networks were assessed by estimating graph‐theoretic parameters, as follows (Y. Wang et al. 2020): (1) Pearson's correlation coefficient was computed for each pair of signals across the 48 CHs to generate a 48 × 48 correlation matrix; (2) correlation values within the correlation matrix were transformed using the Fisher *z*‐transformation to obtain a corresponding *z*‐score matrix; (3) negative values in the *z*‐score matrix were set to zero to sparsify the matrix; (4) the sparsified matrix was converted into a binary matrix, with non‐zero values assigned as 1; (5) graph‐theoretic parameters were calculated for the binary network, including degree centrality, clustering coefficient, and shortest path length; (6) 10 different sparsity thresholds varying from 5% to 50% in increments of 5% were used to mitigate bias associated with the selection of sparsity thresholds; (7) graph‐theoretic parameters were computed for each sparsity threshold, and (8) the area under the curve for the 10 graph‐theoretic parameters was determined using Euler's approximation, which served as a representative index of the graph‐theoretic parameters.

#### 2.3.5 Graph‐Theoretic Parameters

The node degree reflects the significance of each node within a network and is quantified by calculating the number of edges directly connected to a given node, with a larger number of edges indicating a more important node within the network (Bullmore and Bassett 2011).

The clustering coefficient quantifies the extent of network transitivity (i.e., the probability that neighbors of a node are linked). The clustering coefficient reflects the network's capacity for local information transmission as well as the robustness of the network structure. The network clustering coefficient, representing the average of the clustering coefficients across all nodes within the network, was calculated using the following formula:

$$C=\frac{1}{N}\;\sum_{i\in V}\frac{2e_{i}}{k_{i}\left(k_{i}-1\right)}$$

where *N* represents the total number of nodes in the network; *V* denotes the set of nodes within the network; *e_i_* signifies the number of edges connecting the neighbors of node *i*; and *k_i_* (*k_i_* − 1)/2 is the maximum number of potential edges that can be connected to node *i*.

The shortest path length characterizes the optimal route from one node to all other nodes within the network. The network's shortest path length metric represents the average shortest path length between any two nodes and assesses the efficiency of information transmission within the network, with a shorter optimal path corresponding to a higher speed of information transfer (J. Wang et al. 2010). The network's shortest path length was calculated using the following formula:

$$L=\frac{1}{N\left(N-1\right)}\;\sum_{i,j\in V,\;i\neq j}l_{ij}$$

where *l_ij_* denotes the shortest path length between node *i* and node *j*.

### 2.4 Statistical Analyses

Normality of continuous variables was assessed using the Shapiro–Wilk test. Variables without significant deviation from normal distribution, including VFT scores and brain network parameters, were expressed as mean ± standard deviation and compared using an independent‐samples *t*‐test. Non‐normally distributed continuous data were presented as the median and interquartile range and compared using the Mann–Whitney *U* test. Categorical data were reported as numbers with frequencies, which were compared using the chi‐square or Fisher's exact test.

Differences in the degrees of nodes across various CHs between the patient and control groups were examined using a mixed‐design analysis of variance (ANOVA) model. The inter‐group variable was the group (patient vs. control group), and the intra‐group variable was the 48 fNIRS CHs, with the dependent variable being the degree of nodes in each CH. When significant interactions between the inter‐ and intra‐group variables were detected using the ANOVA model, Tukey's post hoc test for multiple comparisons was conducted to identify CHs exhibiting significant differences, with *p* < 0.05 representing statistical significance.

To investigate the associations between brain activities and cognitive decline in the patient group, Pearson's correlation analysis was used to assess the relationships between MMSE scores and (1) differential CHs for task activation, (2) average resting‐state functional connectivity, and (3) graph‐theoretic parameters. *p* values were corrected using the false‐discovery rate (FDR) method for multiple comparisons.

## 3 Results

### 3.1 Clinical Characteristics and Task Performance

The present study included 29 patients with acute CO poisoning (patient group) and 28 healthy controls (control group). There were no significant differences in sex, age, and educational length between the patient and control groups (all *p* > 0.05). The VFT score in the patient group was significantly lower than that of the control group (*t* = 2.35, *p* = 0.023, Cohen's *d *= 0.64) (Table 1). In the patient group, 27 patients completed both tests (VFT and resting‐state), one patient completed only the VFT, and one patient completed only the resting‐state.

**TABLE 1:** Participant characteristics.

Characteristics	Patients (*n* = 29)	Controls (*n* = 28)	*p*
Sex, male/female	15/14	14/14	1.000
Age, year	38.66 (±15.8)	34.93 (± 12.66)	0.334
Length of education, year	14.17 (± 2.74)	14.39 (± 3.31)	0.787
VFT score	11.64 (± 6.03)	15.36 (± 4.61)	0.031
MMSE	23.62 (± 4.51)	—	—
CO exposure duration, (h)	2.00 (1, 10)	—	—
Duration of coma, (h)	0.50 (0, 10)	—	—
COHb saturation (%)	25.03 (± 8.92)	—	—

*Note*: Except for sex, all other data are presented as mean (± standard deviation) or median (interquartile range).

Abbreviations: CO, carbon monoxide; COHb, carboxyhemoglobin; MMSE, Mini‐Mental State Examination; VFT, verbal fluency task.

### 3.2 Comparisons of Task‐Related Brain Activities During VFT Between CO Poisoning Patients and Healthy Controls

Activation of the frontotemporal lobes was compared between the patient and control groups during the VFT. There was a significant difference in the centroid values of the Oxy‐Hb concentration curve in CH26 (*p* < 0.05, FDR‐corrected). Additionally, there were significant differences in the mean activation values of the Oxy‐Hb concentration curves in CH5 and CH6 (both *p* < 0.05, FDR‐corrected) (Figure 1).

**FIGURE 2:**
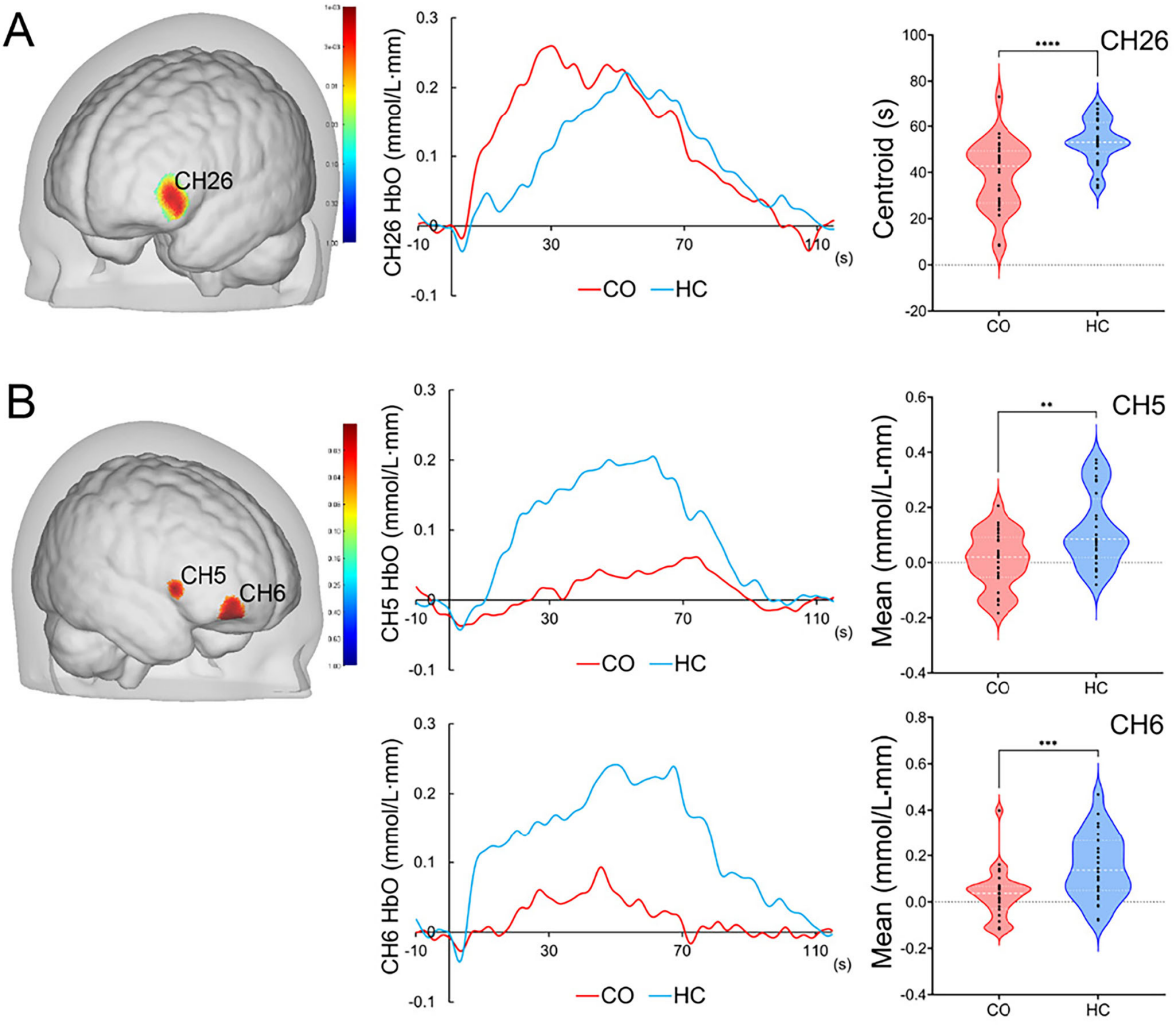
CHs with distinct eigenvalues of brain activation during the VFT. (A) Comparisons of the locations and centroid values for CH26 (****p < 0.0001). (B) Comparisons of the locations and centroid values for CH5 and CH6 (**p < 0.01, ***p < 0.001). CH, channel; VFT, verbal fluency task.

The centroid value of CH26 was significantly lower in the patient group than in the control group (*p* = 0.001, FDR‐corrected), suggesting earlier activation of CH26 in patients with acute CO poisoning. In addition, the mean values for CH5 and CH6 activation were lower in the patient group than in the control group (*p* = 0.017, FDR‐corrected), indicating diminished activation of CH5 and CH6 in patients with acute CO poisoning (Figure 2).

### 3.3 Comparisons of Brain Spontaneous Activities Between CO Poisoning Patients and Healthy Controls

**FIGURE 3:**
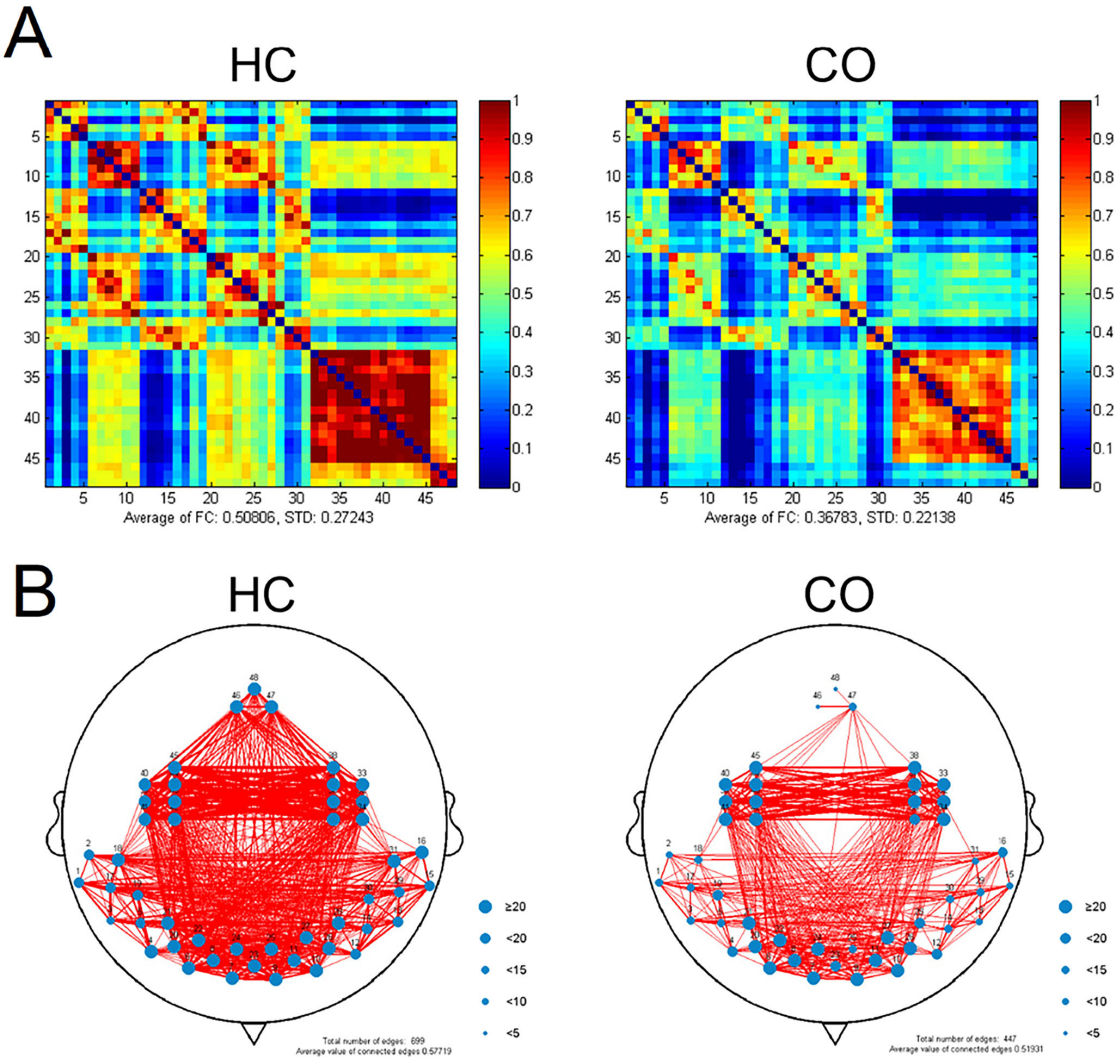
Functional connectivity of each CH across the whole brain. (A) Matrix representing the functional connectivity of the whole brain. (B) CHs exhibiting functional connectivity strength > 0.4. CH, channel.

**FIGURE 4:**
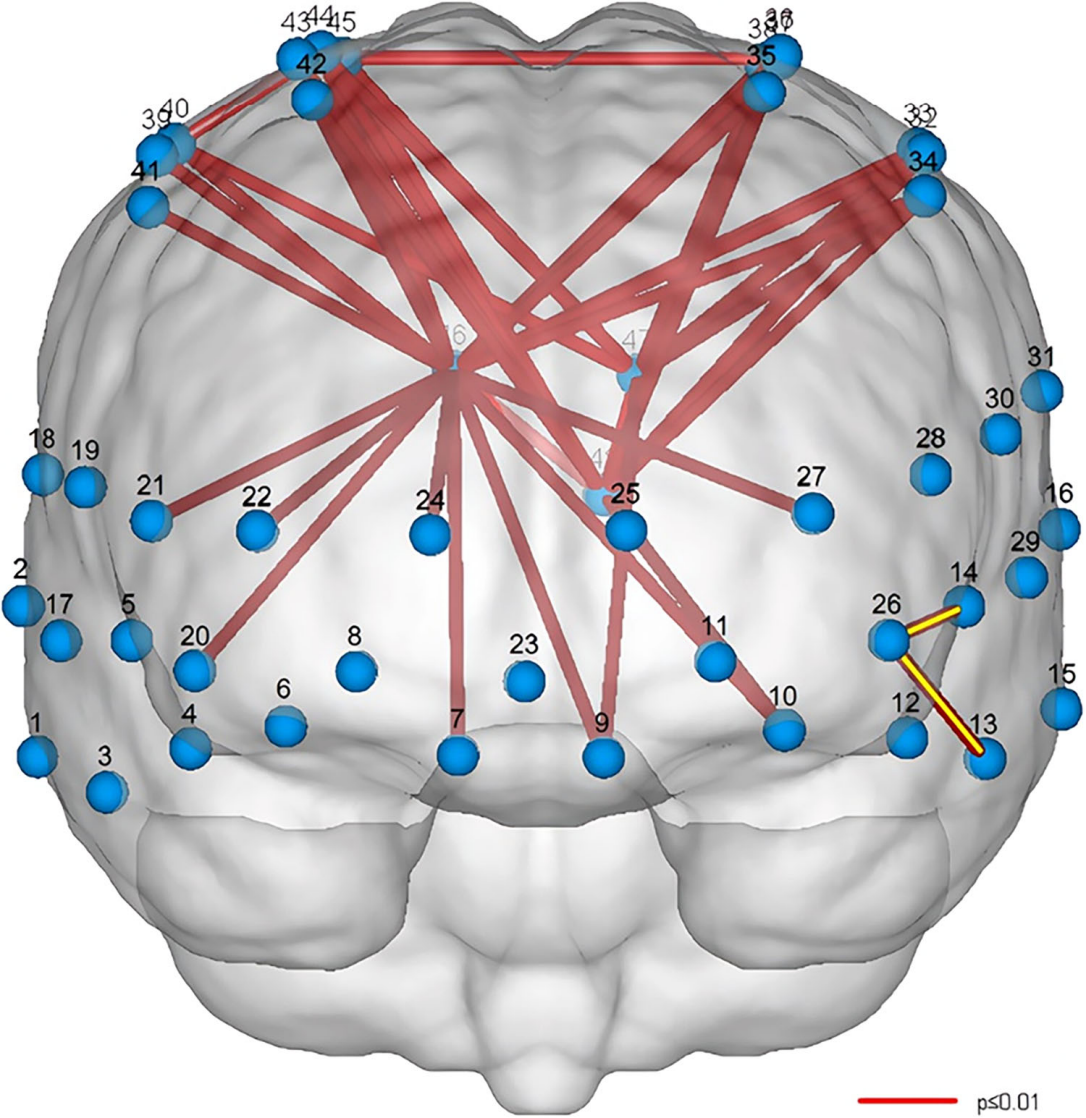
CH pairs with significantly different functional connectivity strengths between the patient and control groups (*p* < 0.01). The two pairs (CH13–CH26 and CH14–CH26) are highlighted in yellow. CH, channel.

The average whole‐brain functional connectivity strength (FCS) was significantly lower in the patient group than in the control group (0.368 ± 0.221 vs. 0.508 ± 0.272; *p* = 0.023, Figure 3). A comparison of the FCS across CHs in the whole brain between the two groups revealed significant differences in the 39 CH pairs (*p* < 0.01, FDR‐corrected). Notably, there were significant disparities in FCS between CH13 and CH26 and between CH14 and CH26 between the two groups (both *p* < 0.01, Figure 4), suggesting that alterations in left dorsolateral prefrontal cortex (dlPFC) activation occurring specific to each group during the VFT might be associated with attenuation of dlPFC‐specific FCS in patients with CO poisoning (note that CH26 corresponds to Brodmann area 46).

**FIGURE 5:**
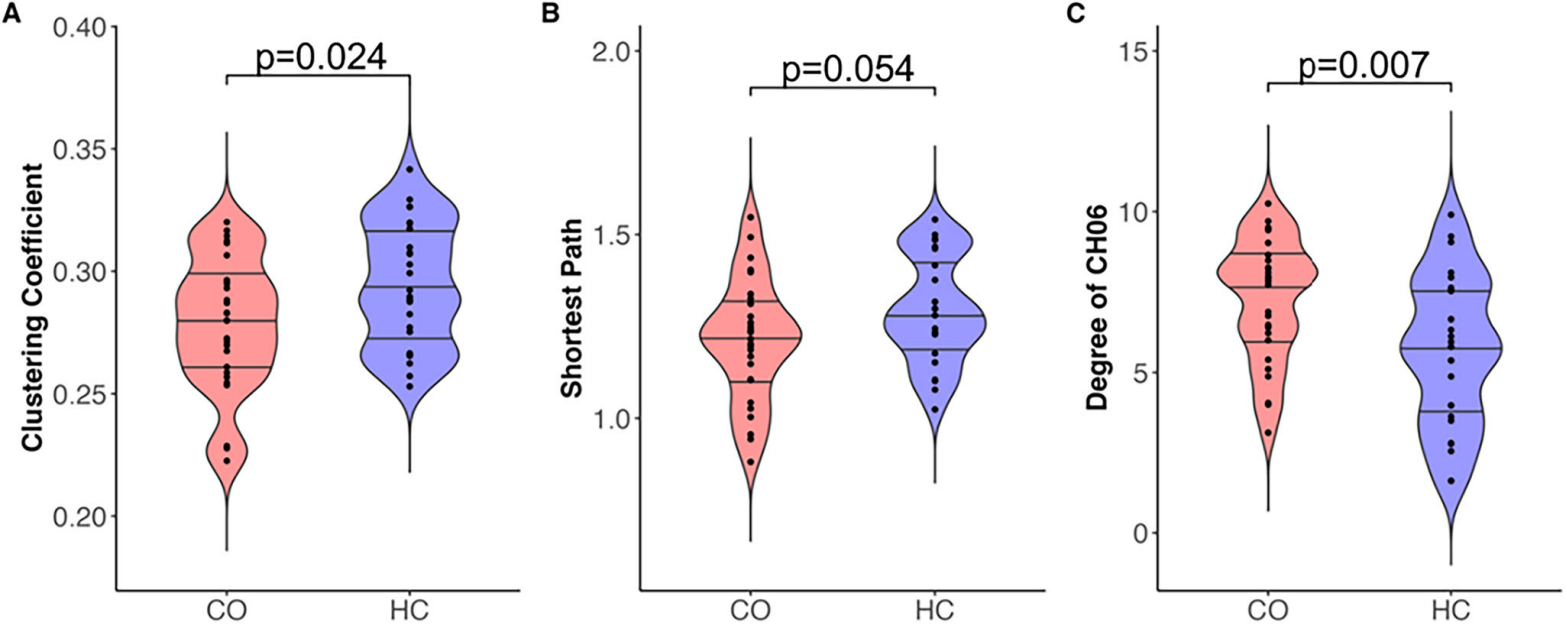
Comparison of differences in graph‐theory parameters between the patient and control groups (*p < 0.05, ***p < 0.001).

**FIGURE 6:**
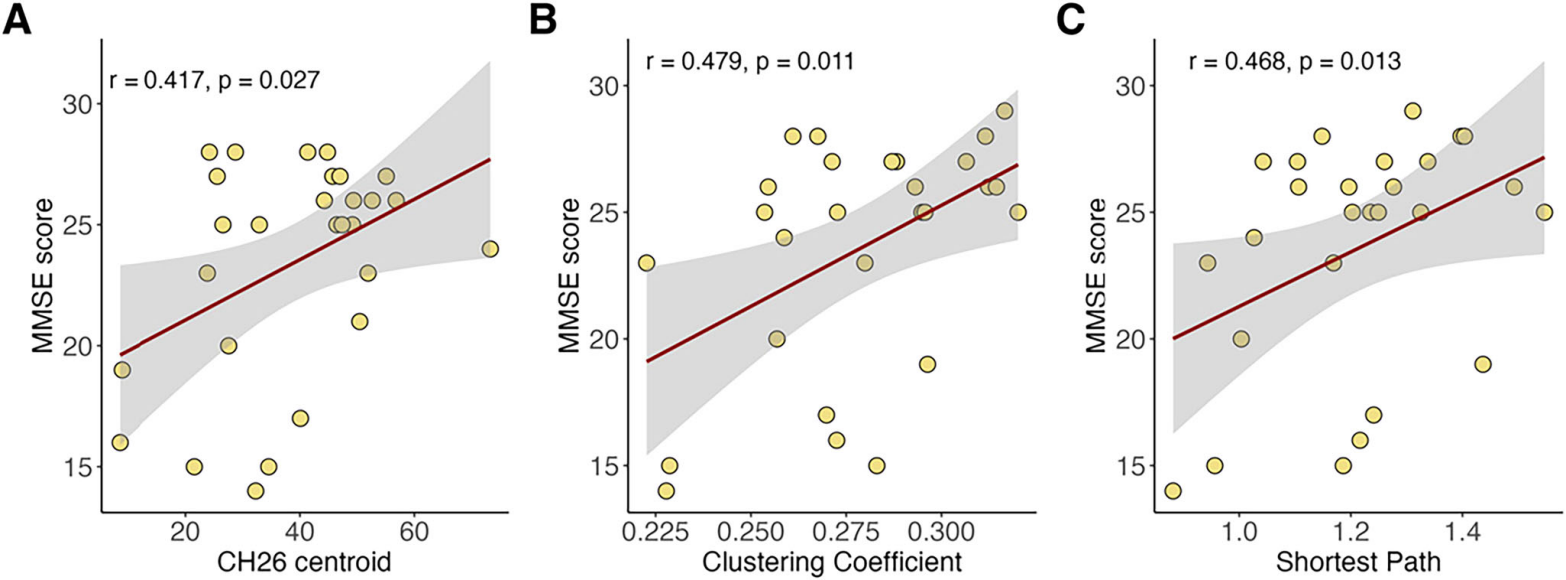
Correlations between brain activities or network indicators and cognitive scores. (A) Centroid values of CH26 during the VFT. (B and C) Relationships between graph‐theoretical parameters and MMSE scores. CH, channel; MMSE, Mini‐Mental State Examination; VFT, verbal fluency task.

We observed a significantly lower network clustering coefficient in the patient group than in the control group (*t* = 2.307, *p *= 0.024). Moreover, differences in the shortest path length between the two groups approached significance (*t* = 1.968, *p* = 0.054). The ANOVA model for the degrees of nodes violated the assumption of sphericity (Mauchly's test *p* < 0.001), and the Greenhouse–Geisser (GG) corrections were applied. After GG corrections, the ANOVA model indicated a significant interaction effect between groups and CHs (*F* [14.1, 761.6] = 2.253, *p* < 0.005). Post hoc multiple comparisons revealed that the degree of nodes in CH6 was significantly higher in the patient group than in the control group *(t* = 2.821, *p* = 0.007). Other CHs that exhibited significant differences included CH8, CH46, CH47, and CH48 (tCH8 = 2.007, tCH46 = −4.613, tCH47 = −2.678, and tCH48 = −3.968, respectively; all *p* < 0.05) (Figure 5). Correlation analysis revealed a significant positive correlation between the centroid value of CH26 and the MMSE score (*r* = 0.417, *p* = 0.027). Moreover, both the network clustering coefficient and the shortest path length exhibited significant positive correlations with the MMSE score (clustering coefficient: *r* = 0.479, *p* = 0.011; shortest path length: *r* = 0.468, *p* = 0.013). However, no significant correlation was observed between the average FCS and the MMSE score (*r* = −0.001, *p* = 0.995). Similarly, the degree of nodes in CH6 was not correlated with the MMSE score (*r* = 0.066, *p* = 0.741, Figure 6).

### 3.4 Comparisons of Treatment‐Related Brain Activity Alterations in CO Poisoning Patients

**FIGURE 7:**
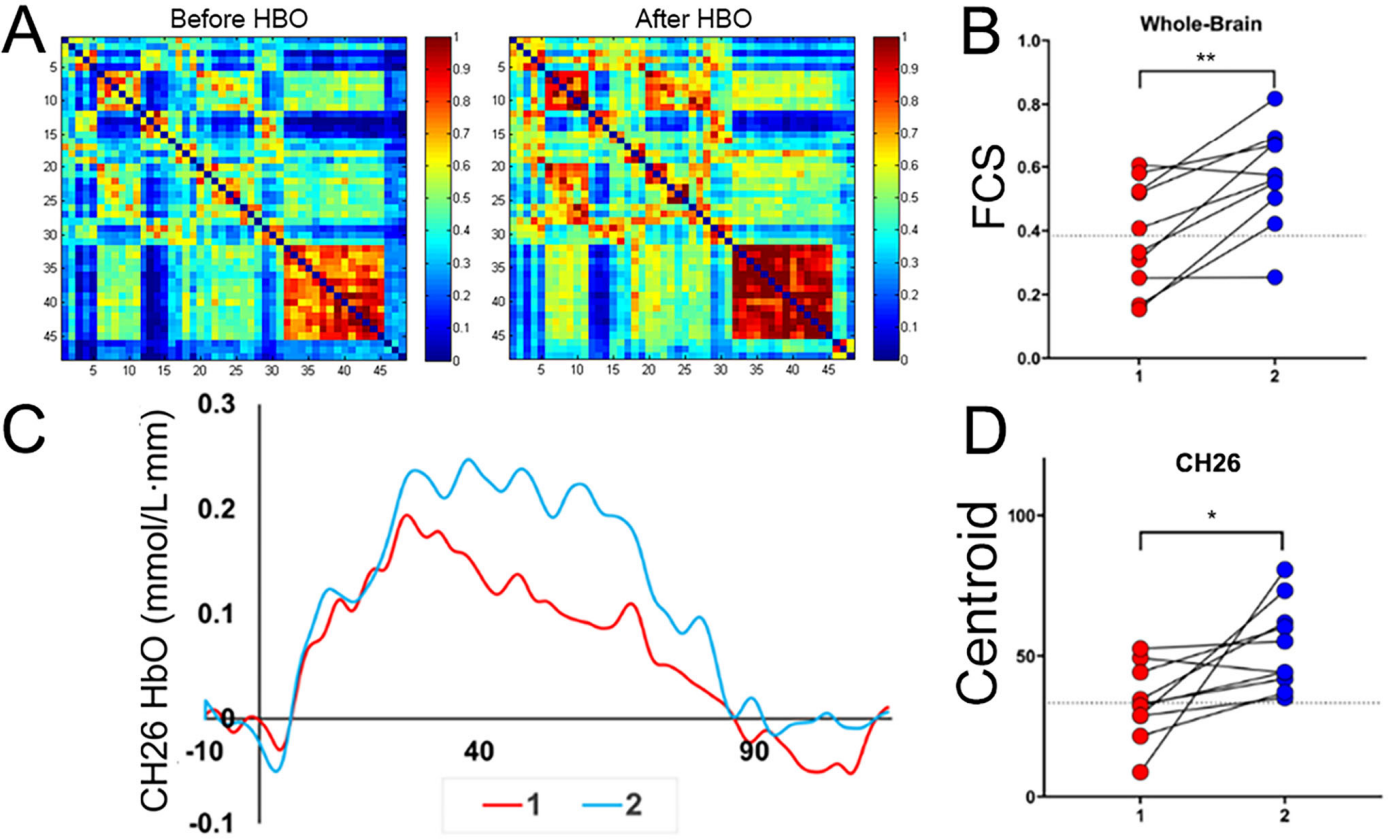
Comparisons of brain functional connectivity strength and activation before and after HBOT. (A) Whole‐brain FCS matrix before and after HBOT. (B) Average whole‐brain FCS before and after HBOT (**p < 0.01); (C) Oxy‐Hb concentration curves for CH29 before and after HBOT. (D) Centroid values of CH26 before and after HBOT (*p < 0.05). CH, channel; HBOT, hyperbaric oxygen therapy; FCS, functional connectivity strength; Oxy‐Hb, oxyhemoglobin.

Ten patients in the patient group underwent a second round of resting‐state and VFT assessments following 20 sessions of HBOT (2.0 atmospheres absolute for 60 min). The average whole‐brain FCS was 0.424 ± 0.228 prior to HBOT, which was significantly increased to 0.503 ± 0.233 after treatment (paired *t*‐test, *p* = 0.002, Figure 7A,B). In the VFT, the centroid values of the Oxy‐Hb concentration curves for CH26 were significantly increased following HBOT (paired *t*‐test, *p* = 0.022, Figure 7C,D).

## 4 Discussion

In the present study, we investigated the brain activity in CO poisoning patients. Through VFT with fNIRS, we found an alteration of brain activities in CO poisoning patients. These brain activity changes were correlated to clinical cognitive performance and could be improved after HBOT.

The abnormality of brain structure and function was reported in previous studies. For instance, T. Wang et al. (2023) found that the CO poisoning patients had pronounced cortical thinning in the bilateral frontoparietal lobes. In another study, Wu, Liu, Zhao, He, and Tan (2020) identified regional homogeneity (ReHo) alteration within bilateral posterior cerebellar lobes, pons, and bilateral basal ganglia, with significantly higher ReHo in the posterior cingulate gyrus, calcarine sulcus, and bilateral occipital lobes in the DEACMP patient. Additionally, MMSE scores in the DEACMP group were negatively correlated with ReHo values in the posterior cingulate gyrus (*r* = −0.672, *p* < 0.05). In a multivariate logistic regression analysis of 387 patients with acute CO poisoning, Jeon et al. (2018) reported that altered mental status, prolonged duration of CO exposure, and the presence of acute brain injury as indicated by diffusion‐weighted imaging were independent risk factors for DEACMP. Of these, acute brain injury detected by diffusion‐weighted imaging had the highest sensitivity and specificity for predicting DEACMP. However, despite the clinical utility of MRI for assessing brain function in patients with CO poisoning, its high costs and inconvenience for behavioral testing have limited its broad clinical application.

fNIRS enables the acquisition of cerebral blood oxygenation signals during the performance of cognitive tasks. It also facilitates the extraction of eigenvalues from Oxy‐Hb curves during the brain activation, thereby offering a quantitative examination and evaluation method for neurological disorders. In the present study, patients with acute CO poisoning exhibited reduced centroid values of Oxy‐Hb curves in the left dlPFC during the VFT. The dlPFC is implicated in various cognitive functions, including working memory, rule learning, planning, attention, and motivation. Moreover, the centroid and mean values of CH26 (Brodmann area 46) were positively correlated with MMSE scores, and its mean value showed a positive correlation with VFT scores. In addition, the mean values of CH5 (Brodmann area 38) and CH6 (Brodmann area 11) were significantly lower in CO poisoning patients than healthy controls. Following 1 month of clinical treatment with HBOT, the centroid values of the Oxy‐Hb curve in the left dlPFC of the patient group were elevated compared with pre‐treatment levels. Thus, the centroid value of the Oxy‐Hb curve in the left dlPFC during the VFT might serve as a characteristic indicator of cognitive function in patients with acute CO poisoning, with potential application as a biomarker for monitoring treatment efficacy.

Functional connectivity refers to the temporal correlation or interdependence of neural activities among spatially isolated neural units (Notte et al. 2024). FCS, which is associated with mental and cognitive activities, serves as an indicator of cognitive function. Jiang et al. (2020) used diffusion tensor imaging to quantify the extent of deep gray matter damage and attempted to analyze the relationships between brain injury and memory or cognitive impairments in patients with acute CO poisoning. They found that hippocampal damage was associated with clinical symptoms and cognitive alterations in acute CO poisoning. A subsequent diffusion tensor imaging study demonstrated that white matter integrity alterations could persist for up to 4–6 months after CO poisoning, even following HBOT, and revealed the correlation between the white matter damage and cognitive impairments (Chang et al. 2009). Furthermore, diminished connectivity resulting from these white matter alterations could offer a pathophysiological basis for the cognitive deficits observed in patients with CO poisoning. H. Liu et al. (2023) investigated the topological alterations in whole‐brain functional connectivity in children with CO poisoning and reported compromised brain network topology, characterized by decreased global efficiency and small‐worldness, as well as increased characteristic path length. These findings collectively suggest the presence of abnormal brain functional connectivity in patients with CO poisoning.

In the present study, the average whole‐brain resting‐state FCS was significantly lower in patients with acute CO poisoning compared to healthy controls. There was also a significant difference in FCS across the 39 CH pairs between the two groups. Furthermore, following 1 month of clinical treatment with HBOT, the average whole‐brain FCS was increased compared with the pre‐treatment levels in the patient group. These findings implied that the average whole‐brain FCS in patients with acute CO poisoning could be applied to serve as a biomarker for monitoring treatment efficacy. In addition, a recent fNIRS study showed that the patients with DEACMP, representing a poor outcome after acute CO poisoning, exhibited abnormal functional connectivity in both whole‐brain and cognitive‐related key brain regions (Yang et al. 2025). This further supported our findings that whole‐brain FCS can be used to predict prognosis after acute CO poisoning. Interestingly, however, no significant correlation was observed between the average FCS and MMSE scores, possibly due to the following reasons: (1) the relatively mild acute CO poisoning among most patients in the present study; (2) the limited reliability and sensitivity of cognitive screening methods used for detecting subtle alterations in cognitive function; and (3) our relatively small sample size.

There are several limitations in our study that should be acknowledged. First, this study was conducted in an urban setting where the number of eligible CO poisoning cases was relatively limited, resulting in a modest sample size. Although a medium effect size was observed for the primary outcome, the statistical power was moderate (post hoc power = 0.652). Therefore, our findings should be interpreted with caution and considered exploratory, pending validation in larger multicenter studies. Second, brain function changes were evaluated in patients after CO poisoning. The post‐poisoning changes could have been impacted by baseline pre‐poisoning status, which was not available for analysis. Finally, we showed brain activity changes after the HBOT. However, whether pre‐treatment brain activity changes could predict treatment response and long‐term prognosis requires further studies.

## 5 Conclusions

In patients with acute CO poisoning, the centroid of the Oxy‐Hb concentration curve in the left dlPFC during the VFT and the average whole‐brain resting‐state FCS could be applied to assess brain function. In addition, the centroid values of the Oxy‐Hb concentration curve during the VFT are a potential biomarker for assessing treatment response in patients with acute CO poisoning, thereby offering a foundation for personalized treatment strategies.

## Author Contributions

**Jian‐Qing Pan**: methodology, resources, writing – original draft, conceptualization, investigation. **Ming‐Xing Tang**: writing – review and editing. **Lian Duan**: writing – review and editing. **Qing‐Ping Zhang**: writing – review and editing, funding acquisition. **Qun‐Jun Liang**: methodology, writing – review and editing.

## Ethics Statement

This study was approved by the Shenzhen Nanshan People's Scientific Research Ethics Committee (Approval number: ky‐2024‐031702, ky‐2025‐0828002).

## Consent

All participants in the experiments gave informed consent to participate in this study.

## Conflicts of Interest

The authors declare no conflicts of interest.

## Data Availability

The dataset and codes are available upon reasonable request to the corresponding author.
